# Carboplatin binding to histidine

**DOI:** 10.1107/S2053230X14016161

**Published:** 2014-08-29

**Authors:** Simon W. M. Tanley, Kay Diederichs, Loes M. J. Kroon-Batenburg, Colin Levy, Antoine M. M. Schreurs, John R. Helliwell

**Affiliations:** a School of Chemistry, Faculty of Engineering and Physical Sciences, University of Manchester, Brunswick Street, Manchester M13 9PL, England; b Department of Biology, University of Konstanz, D-78457 Konstanz, Germany; c Crystal and Structural Chemistry, Bijvoet Center for Biomolecular Research, Faculty of Science, Utrecht University, Padualaan 8, 3584 CH Utrecht, The Netherlands; d Manchester Institute of Biotechnology (MIB), University of Manchester, 131 Princess Street, Manchester M1 7DN, England

**Keywords:** carboplatin, histidine, avoid partial conversion to cisplatin, NaBr crystallization conditions, non-NaCl crystallization conditions, model protein (hen egg-white lysozyme)

## Abstract

Carboplatin is a second-generation platinum anticancer agent used for the treatment of a variety of cancers. Previous X-ray crystallographic studies of carboplatin binding to histidine (in hen egg-white lysozyme; HEWL) showed the partial conversion of carboplatin to cisplatin owing to the high NaCl concentration used in the crystallization conditions. HEWL co-crystallizations with carboplatin in NaBr conditions have now been carried out to confirm whether carboplatin converts to the bromine form and whether this takes place in a similar way to the partial conversion of carboplatin to cisplatin observed previously in NaCl conditions. Here, it is reported that a partial chemical transformation takes place but to a transplatin form. Thus, to attempt to resolve purely carboplatin binding at histidine, this study utilized co-crystallization of HEWL with carboplatin without NaCl to eliminate the partial chemical conversion of carboplatin. Tetragonal HEWL crystals co-crystallized with carboplatin were successfully obtained in four different conditions, each at a different pH value. The structural results obtained show carboplatin bound to either one or both of the N atoms of His15 of HEWL, and this particular variation was dependent on the concentration of anions in the crystallization mixture and the elapsed time, as well as the pH used. The structural details of the bound carboplatin molecule also differed between them. Overall, the most detailed crystal structure showed the majority of the carboplatin atoms bound to the platinum centre; however, the four-carbon ring structure of the cyclobutanedicarboxylate moiety (CBDC) remained elusive. The potential impact of the results for the administration of carboplatin as an anticancer agent are described.

## Abstract 2

An X-ray crystal structure showing the binding of purely carboplatin to histidine in a model protein has finally been obtained. This required extensive crystallization trials and various novel crystal structure analyses.

## 1. Introduction   

**Figure 1:**
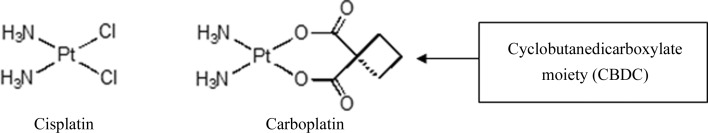
Chemical diagrams of cisplatin and carboplatin.

Cisplatin and carboplatin are platinum anticancer drugs which have long been used in the fight against cancer by targeting DNA. However, 90% of their reported binding cases are to plasma proteins (Fischer *et al.*, 2008 ▶). Thus, these drugs cause toxic side effects. Cisplatin is rapidly converted to toxic metabolites which have nephrotoxic effects (Zhang & Lindup, 1996 ▶; Huličiak *et al.*, 2012 ▶), whereas carboplatin is less toxic owing to the addition (Fig. 1 ▶) of the cyclobutanedicarboxylate moiety (CBDC), which has a slower rate of conversion. Carboplatin can therefore be tolerated by patients at higher doses compared with cisplatin (Kostova, 2006 ▶).

The crystal structure of carboplatin on its own has been determined (Beagley *et al.*, 1985 ▶), in which one of the cyclobutane C atoms showed excessive thermal motion, which was interpreted in terms of a dynamically puckering ring. We have built on this work and on the study of Casini *et al.* (2007 ▶), which reported the crystal structure of cisplatin with hen egg-white lysozyme (HEWL), showing one cisplatin bound to His15. Casini *et al.* (2007 ▶) also reported mass-spectrometric data for both cisplatin and carboplatin binding to HEWL. Structures of cisplatin binding to the histidine residues of superoxide dismutase (Calderone *et al.*, 2006 ▶; Casini *et al.*, 2008 ▶) and of cytochrome *c* (Casini *et al.*, 2006 ▶) have also been determined. Our X-ray crystallographic studies of cisplatin with HEWL, a model protein, have shown binding of two molecules to its His15 residue in dimethyl sulfoxide (DMSO) medium (Tanley, Schreurs, Kroon-Batenburg & Helliwell, 2012 ▶; Tanley, Schreurs, Kroon-Batenburg, Meredith *et al.*, 2012 ▶; Helliwell & Tanley, 2013 ▶) and even after prolonged exposure in an aqueous medium without DMSO (Tanley, Schreurs, Kroon-Batenburg & Helliwell *et al.*, 2012 ▶). Subsequently, through public archiving of our raw diffraction images at Utrecht University (Tanley, Schreurs *et al.*, 2013 ▶; http://rawdata.chem.uu.nl/#0001; http://rawdata.chem.uu.nl/#0002), now also mirrored at the Tardis Raw data archive in Australia (http://vera183.its.monash.edu.au/experiment/view/40/), a collaboration was set up with one of the authors of this article (KD), who downloaded and reprocessed the diffraction images measured at the University of Manchester with the *XDS* software package (Kabsch, 2010 ▶) to compare with our previous results. Reviewing those results along with our previous publication of the carboplatin-bound structures in DMSO medium studied at cryo and room temperatures (Tanley, Schreurs, Kroon-Batenburg & Helliwell, 2012 ▶; Tanley, Schreurs, Kroon-Batenburg, Meredith *et al.*, 2012 ▶; PDB entries 4dd7, 4dd9 and 4g4c), it was noted that there were two small extra anomalous difference electron-density peaks within the carboplatin binding sites (Tanley, Diederichs *et al.*, 2013 ▶ *a*). This suggested that in the high NaCl concentrations used in our crystallization conditions (Tanley, Schreurs, Kroon-Batenburg & Helliwell, 2012 ▶; Tanley, Schreurs, Kroon-Batenburg, Meredith *et al.*, 2012 ▶; Helliwell & Tanley, 2013 ▶) carboplatin could be partially converted to cisplatin, with the extra anomalous difference density thereby being attributed to the Cl atoms of cisplatin. This partial conversion of carboplatin had been observed previously in solution (Gust & Schnurr, 1999 ▶). Owing to these new findings, the His15 binding sites could thus contain a mixture of carboplatin and cisplatin rather than just the pure carboplatin molecule.

Based on these findings (Tanley, Diederichs *et al.*, 2013*a* ▶) arising from sharing the raw diffraction data images, we now report the co-crystallization of HEWL and carboplatin in NaBr conditions, in which the expected two bromines should be more readily visible than the two partially occupied chlorines. We also have investigated the crystallization of HEWL with carboplatin in non-salt, *i.e.* neither NaCl nor NaBr, conditions to completely remove the possibility of carboplatin converting to the chloro or bromo forms. Again, we were able to build on a very useful previous study (Weiss *et al.*, 2000 ▶) which crystallized HEWL in 75% MPD at pH 8.0. In addition, we made an extensive search whereby we surveyed 48 different non-NaCl crystallization conditions from Hampton Research (Supplementary Table S1^1^) with the aim of finding a variety of conditions and pH values in order to determine which gave the best detailed binding site of the carboplatin molecule in the absence of these salt ions in the crystallization mixture. We have also carried out an elemental analysis of the Sigma-supplied HEWL to scrutinize any chloride content.

## 2. Methods   

### 2.1. Crystallization conditions   

#### 2.1.1. NaBr   

Co-crystallization of HEWL with carboplatin in NaBr solution was carried out under similar conditions as published by Dauter & Dauter (1999 ▶) and Lim *et al.* (1998 ▶) but co-crystallizing 20 mg ml^−1^ HEWL with 1.4 mg carboplatin in 75 µl DMSO, 462.5 µl 0.1 *M* sodium acetate, 462.5 µl 1 *M* NaBr solution.

#### 2.1.2. Conditions without NaCl or NaBr   

20 mg HEWL (0.6 m*M*) was dissolved in 1 ml distilled water. 1.4 mg carboplatin (1.8 m*M*) was added in a threefold molar excess over the protein along with 75 µl DMSO and was mixed until all of the carboplatin had dissolved. 48 crystal screens from Hampton Research (listed in Supplementary Table S1) were set up; these comprised 2 µl protein/carboplatin/DMSO solution aliquots each mixed with 2 µl reservoir solution and were set up as hanging-drop crystallizations with 1 ml reservoir solution. The crystallization trays were left at room temperature and the crystals that yielded detailed structural results, as described below, grew in the conditions (i) 65% MPD with 0.1 *M* citric acid buffer at pH 4.0, (ii) 0.2 *M* ammonium sulfate, 0.1 *M* sodium acetate in 25% PEG 4000 at pH 4.6, (iii) 0.1 *M* sodium citrate, 20% propanol, 20% PEG 4000 at pH 5.6 and (iv) 2 *M* ammonium formate, 0.1 *M* HEPES at pH 7.5.

#### 2.1.3. Other non-NaCl or NaBr conditions   

In addition to those described in §[Sec sec2.1.2]2.1.2, crystals also grew in the following conditions: (i) 0.1 *M* imidazole, 1 *M* sodium acetate pH 4.6, (ii) 20% Jeffamine 500, 0.1 *M* HEPES pH 7.5 and (iii) 0.1 *M* Na HEPES, 0.8 *M* sodium potassium tartrate. These showed that carboplatin had not bound.

#### 2.1.4. Elemental analysis of the HEWL lyophilized powder from Sigma   

Elemental analysis scrutinizing the chloride content of the lyophilized HEWL powder purchased from Sigma showed that there was 2.6% chlorine present. For crystallization, 20 mg HEWL was dissolved in 1 ml water and 2 µl aliquots of this solution were used to set up the hanging-drop crystallizations. Thus, from the starting 2.6% chlorine, the percentage of chlorine in each of our crystallization droplet conditions was around 0.005%. This is therefore much lower than the 10% (1.4 *M*) NaCl solution used in our previous crystallization conditions (Tanley, Schreurs, Kroon-Batenburg & Helliwell, 2012 ▶; Tanley, Schreurs, Kroon-Batenburg, Meredith *et al.*, 2012 ▶; Helliwell & Tanley, 2013 ▶), indicating that there would be no significant conversion to cisplatin.

### 2.2. X-ray diffraction data collection, protein structure solution and model refinement   

Crystals were each scooped into a loop using silicone oil as a cryoprotectant. All X-ray diffraction (XRD) data were measured on a Bruker APEX II home-source diffractometer at an X-ray wavelength of 1.5418 Å, except for one of the NaBr-grown crystals, from which XRD data were collected on beamline I04 at Diamond Light Source (DLS) with an X-ray wavelength of 0.9163 Å; namely, the short-wavelength side of the Br *K* edge. The XRD data collections were carried out at fixed temperatures between 100 and 127 K (Table 1 ▶). For the home laboratory runs the XRD data from each crystal were processed using the Bruker software package *SAINT* (Bruker AXS, Madison, WI, USA), with the exception of the crystal from the 65% MPD with 0.1 *M* citric acid buffer at pH 4.0 crystallization mixture, for which one data set was processed with *EVAL* (Schreurs *et al.*, 2010 ▶) and the other with *XDS* (Kabsch, 2010 ▶). The data for the NaBr-grown crystal XRD data set collected on beamline I04 at DLS were processed with *MOSFLM* (Leslie, 1999 ▶).

**Figure 2:**
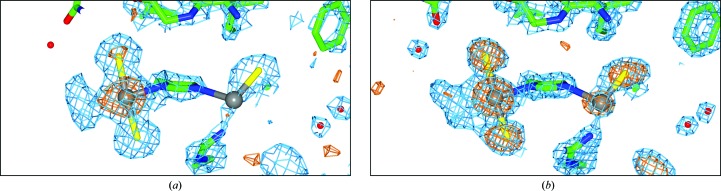
The His15 binding site for platinum with Br atoms as labelled assigned as bound to Pt in the *trans* conformation owing to the presence of anomalous difference electron density. We have placed atoms where we are confident of their assignment, namely the histidine, the bromines and the platinums. At the extreme left the density is less easily interpretable. (*a*) The Cu *K*α data set and (*b*) the I04 data set from Diamond. The 2*F*_o_ − *F*_c_ map (blue) is shown at the 1.5 r.m.s. contour level and the anomalous difference electron-density map (orange) is shown at the 3σ contour level. The Pt atoms are shown in grey, the Br atoms are shown in yellow, C atoms are in green, O atoms are in red and N atoms are in blue.

**Figure 3:**
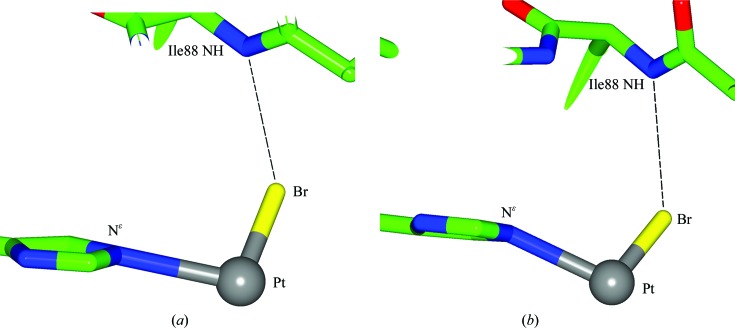
The distances between atoms in the N^∊^ binding site of the crystals grown in NaBr conditions. (*a*) The Cu *K*α data set and (*b*) the I04 Diamond data set. (*a*) The Pt—N^∊^ bond distance is 2.5 ± 0.6 Å, the Pt—Br distance is 2.4 ± 0.5 Å and the Br to NH distance is 3.6 ± 0.4 Å. (*b*) The Pt—N^∊^ bond distance is 2.6 ± 0.1 Å, the Pt—Br distance is 2.5 ± 0.1 Å and the Br to NH distance is 3.5 ± 0.1 Å. The sigmas given on the bond distances, which were unrestrained, were calculated using the Cruickshank DPI values (Cruickshank, 1999 ▶). The distance between the Br atom and the NH group of Ile88 is a typical halide hydrogen-bond distance. The Pt atom is shown in grey, the Br atom in yellow, C atoms are in green, O atoms are in red and N atoms are in blue.

**Figure 4:**
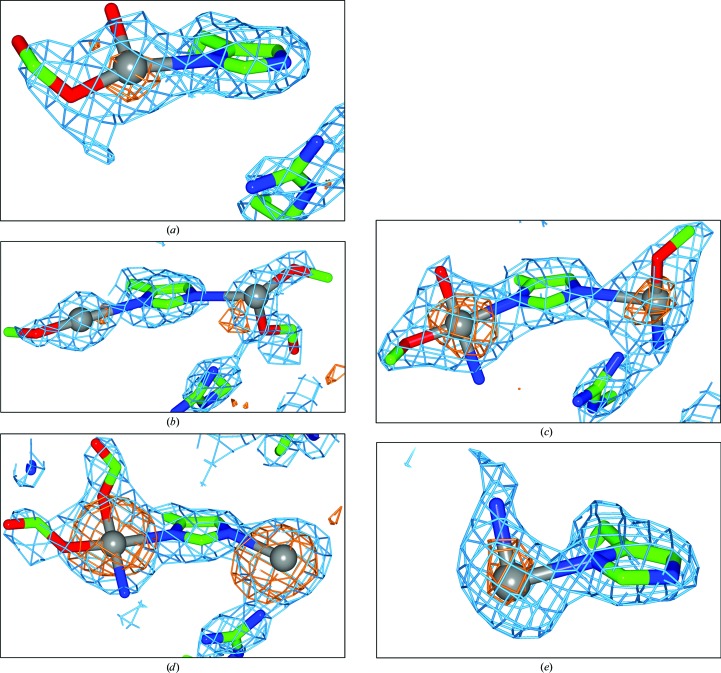
Binding of carboplatin to the N^δ^ and/or N^∊^ atom of His15. (*a*) 65% MPD, 0.1 *M* citric acid buffer pH 4.0 crystal 1, (*b*) 65% MPD, 0.1 *M* citric acid buffer pH 4.0 crystal 2, (*c*) 0.2 *M* ammonium sulfate, 0.1 *M* sodium acetate, 25% PEG 4000 pH 4.6, (*d*) 0.1 *M* sodium citrate, 20% propanol, 20% PEG 4000 pH 5.6, (*e*) 2 *M* ammonium formate, 0.1 *M* HEPES pH 7.5. 2*F*_o_ − *F*_c_ maps (blue) are shown at the 1.2 r.m.s. contour level. Anomalous difference electron-density (orange) maps are shown at the 3.0σ contour level. The Pt atoms are shown in grey, C atoms are in green, O atoms are in red and N atoms are in blue.

The crystal structures were solved using molecular replacement with *Phaser* (McCoy *et al.*, 2007 ▶), using the reported lysozyme structure with PDB code 2w1y as a molecular search model (Cianci *et al.*, 2008 ▶) and restrained refinement with *REFMAC*5 (Murshudov *et al.*, 2011 ▶) from *CCP*4 (Winn *et al.*, 2011 ▶). For the NaBr-grown crystal for which data were collected on beamline I04 at DLS, anisotropic atomic *B* factors were refined, as the high resolution afforded this possibility, whereas all other data sets were refined with isotropic atomic *B* factors. Model building, adjustment and refinement were carried out using *Coot* (Emsley *et al.*, 2010 ▶) and *REFMAC*5 in *CCP*4. Ligand-binding occupancies were refined using *SHELXTL* (Sheldrick, 2008 ▶). The crystallographic and molecular model-refinement parameters are summarized in Table 1 ▶. The e.s.d. values for the Pt occupancies (Table 2 ▶) were refined using the full-matrix inversion technique in *SHELXL* (Sheldrick, 2008 ▶). For resolutions worse than 2.5 Å refined occupancy estimates become questionable. Figs. 2, 3, 4, S1 and S2 were prepared with *CCP4mg* (McNicholas *et al.*, 2011 ▶).

## 3. Results   

### 3.1. Carboplatin crystallization with HEWL in NaBr crystallization conditions   

#### 3.1.1. The formation of transbromoplatin in the N^δ^ binding site   

This experiment sought to make any conversion to the bromo platinated form more clearly discernible than the chloro version described in Tanley, Diederichs *et al.* (2013*a* ▶). The most surprising result is that the transbromoplatin form occurred rather than a cisbromoplatin form (Fig. 2 ▶); see http://en.wikipedia.org/wiki/File:Cisplatin_and_transplatin.gif, which shows an explicit comparison. Two crystals were studied; each was studied quite a long time after crystallization had been set up, namely after ten and 11 weeks.

For the crystal from which the Cu *K*α data set was collected, platinum binding is seen at both the N^δ^ and N^∊^ binding sites (Fig. 2 ▶ *a*) with anomalous difference electron-density peak heights for the Pt positions of 12.1σ and 3.2σ observed in the N^δ^ and N^∊^ binding sites, respectively, which agree closely with the respective Pt occupancy values of 94% (±6%) and 28% (±7%) refined by *SHELXL* (Table 2 ▶). An occupancy value of 94% is the highest that we have observed for a Pt atom in any cisplatin, carboplatin or, now, transbromoplatin form. The N^∊^ binding site is harder to interpret owing to there being two (and not three) anomalous difference density peaks; they are both weak and they are of similar heights. The interpretation (shown in Figs. 2 ▶ *a* and 3 ▶ *a*) is guided by the expected distance to His15 N^∊^ of the Pt and the distance of the peak assigned as bromine to this Pt. A second crystal was used to collect the XRD data set for the NaBr condition, which was collected on beamline I04 at Diamond Light Source using an X-ray wavelength of 0.9163 Å, thereby on the short-wavelength side of the Br *K* edge and with an optimized Br *f*′′ signal (3.9 *versus* 1.4 electrons at Cu *K*α). Fig. 2 ▶(*b*) shows the difference electron-density map. Similarly to the Cu *K*α data-set results described above, a Pt atom is seen bound to both the N^δ^ and N^∊^ atoms of His15, again with a strongly occupied Pt in the N^δ^ binding site (76 ± 12%) and a weak occupancy in the N^∊^ binding site (13 ± 11%). In the N^δ^ binding site, besides the Pt peak, there are two large anomalous difference density peaks of 13σ and 15σ which are readily assignable as Br atoms and are at distances of 2.5 Å (±0.1 Å) from the Pt atom, definitely confirming that the carboplatin has converted to the transbromoplatin form. These Br atoms are also confirmed by the presence of strong 2*F*_o_ − *F*_c_ electron-density peaks. In the N^∊^ binding site, where there is weak binding, the interpretation is still difficult but is easier owing to the strong anomalous difference map peaks compared with the Cu *K*α case. The Pt atom could be assigned based on the closest distance to the His15 N^∊^ atom, and the Br peak assignment then naturally followed (Fig. 3 ▶ *b*).

A recent study of an iodo form of cisplatin bound to His15 of HEWL (Messori *et al.*, 2013 ▶) showed ‘peculiar features’ involving

the presence of three peaks of anomalous electron density close to a Pt atom suggesting the presence of two alternative mode of binding of the [PtI_2_NH_3_] moiety.

The 2*F*_o_ − *F*_c_ density at the extreme left of the N^δ^ Pt centre in the Cu *K*α and the I04 Diamond data sets has a very similar shape, and also there is no anomalous difference electron density, ruling out the possibility of a Br atom substituting at this third position. This electron density is also too detailed to be a single N atom. Thus, a portion of the CBDC moiety of the carboplatin molecule (Fig. 1 ▶) must still be present at this position, suggesting that the carboplatin has ‘only’ partially converted to the transbromoplatin form.

#### 3.1.2. Other bromine binding sites   

Based on the anomalous difference electron-density map, it is observed that Br atoms are indeed bound to the protein and are in the same positions as observed by Dauter & Dauter (1999 ▶) and Lim *et al.* (1998 ▶), with these sites also being the same as the usual Cl atom binding sites.

### 3.2. Carboplatin binding to His15 in non-NaCl conditions   

For the crystals grown in 65% MPD, 0.1 *M* citric acid buffer pH 4.0 conditions, XRD data were collected from two separate crystals four weeks apart (Fig. 4 ▶ *a* and 4 ▶ *b*). Data from crystal 1 was collected one week after crystal growth and show carboplatin bound to the N^δ^ binding site of His15 (Fig. 4 ▶ *a*). However, data for crystal 2 from the same crystallization drop were collected five weeks after crystal growth was set up and a molecule of carboplatin is now seen bound to both the N^δ^ and N^∊^ atoms of His15 (Fig. 4 ▶ *b*). These two crystals also show differences in the amount of detail observed for the carboplatin molecule at the N^δ^ binding site. Most importantly, the amount of detail in the N^∊^ binding site of crystal 2 (Fig. 4 ▶ *b*) is the most that we have seen at this particular binding site out of all of the crystals studied, where we observe a portion of the CBDC moiety.

The crystal grown in 0.2 *M* ammonium sulfate, 0.1 *M* sodium acetate, 25% PEG 4000 pH 4.6 shows a molecule of carboplatin bound to both the N^δ^ and N^∊^ atoms of His15 (Fig. 4 ▶ *c*). No extra detail is seen in either binding site compared with the crystals grown at pH 4.0 in 65% MPD described above.

The crystal grown in 0.1 *M* sodium citrate, 20% propanol, 20% PEG 4000 pH 5.6 again shows a molecule of carboplatin bound to both the N^δ^ and N^∊^ atoms of His15 (Fig. 4 ▶ *d*). In the N^∊^ binding site the electron density allows only a Pt atom to be modelled. However, at the N^δ^ binding site in this pH 5.6 crystal, one sees the most detail for the carboplatin molecule, with only the four-carbon ring of the CBDC moiety missing in the electron density. See Supplementary Fig. S1 for a full carboplatin molecule superimposed on the electron density at the His15 binding site. It was from this that those carboplatin atoms that were not in electron density were deleted from the model.

The last condition in which crystals were obtained was 2 *M* ammonium formate, 0.1 *M* HEPES pH 7.5. These crystals only show carboplatin bound to the N^δ^ binding site (Fig. 4 ▶ *e*) and with electron density visible solely for the Pt atom and one N atom, whereas in the N^∊^ binding site 2*F*_o_ − *F*_c_ electron density is seen at 2.5 r.m.s. but no anomalous difference density is present. However, this electron density is around 3.5 Å from the N^∊^ atom, and thus we cannot interpret it as a Pt atom even if it might be weakly bound owing to this unusually large distance (compared with 2.4 Å when properly bound).

The occupancy values of the Pt atoms at each binding site along with the anomalous difference density peak heights are given in Table 2 ▶ for each of the ‘non-NaCl’ crystallization conditions. With the exception of the crystal at pH 5.6, all of the occupancy values are lower compared with the average Pt occupancies seen previously in NaCl crystallization conditions (∼70% for the N^δ^ binding site and ∼50% for the N^∊^ binding site with an estimated ±5% standard uncertainty; Tanley, Schreurs, Kroon-Batenburg, Meredith *et al.*, 2012 ▶).

The pH 7.5 crystal showed two unusual *F*_o_ − *F*_c_ density and anomalous difference density peaks very near to one of the disulfide bonds (Cys6–Cys127; see Supplementary Fig. S2).

## 4. Discussion   

### 4.1. Carboplatin binding to His15 in NaBr co-crystallization conditions   

Using NaBr in the crystallization conditions confirmed the partial chemical conversion of carboplatin, as with NaCl. Here, though, we see a transbromoplatin form rather than chloro cisplatin. The transbromoplatin in the NaBr-grown crystal studied at the Cu *K*α X-ray wavelength has the highest occupancy that we have seen (94 ± 6% at the His15 N^δ^ position).

### 4.2. Carboplatin binding to His15 in non-halide co-crystallization conditions   

#### 4.2.1. The different forms of His15 lead to differing binding modes   

From the four crystallization conditions in which carboplatin is seen bound to the His15 residue, the percentage of the binding of carboplatin molecules to the N^δ^ and N^∊^ atoms of His15 varies considerably. This chemical behaviour can be compared with the NaCl crystallization conditions, which always showed one cisplatin bound at each of the N^δ^ and N^∊^ atoms of His15 (Tanley, Schreurs, Kroon-Batenburg & Helliwell, 2012 ▶; Tanley, Schreurs, Kroon-Batenburg, Meredith *et al.*, 2012 ▶; Helliwell & Tanley, 2013 ▶).

**Figure 5:**
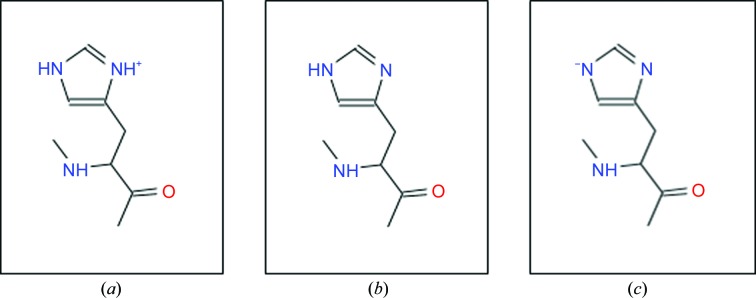
The three different forms of His. (*a*) The protonated form which exists at pH < 6.0, (*b*) the deprotonated form which exists at physiological pH values between 6.5 and 7.5 and (*c*) the imidazolyl anion form which exists once both of the N–H atoms have been extracted.

As we have explained previously (Tanley, Schreurs, Kroon-Batenburg, Meredith *et al.*, 2012 ▶), and as we briefly reiterate here, the case of binding to both the N^δ^ and N^∊^ atoms of His15 could be owing to the His residue being an imidazolyl anion or owing to the histidine being able to exist in two tautomeric forms in solution at a pH similar to the p*K*_a_ (6.0–6.3). In the case of two tautomeric forms, either the N^δ^ or the N^∊^ atom can participate in the interaction with Pt. The imidazolyl anion is formed upon the removal of both of the N–H atoms and can be brought about by a high concentration of Cl^−^ ions (1.4 *M*) in the co-crystallization conditions used in these studies (Tanley, Schreurs, Kroon-Batenburg, Meredith *et al.*, 2012 ▶). The imidazolyl anion has a lone pair of electrons on both N atoms each capable of bonding to metal atoms. Thus, the His residue is known to be able to exist in three different forms: protonated His at pH <6.0, deprotonated His at physiological pHs between 6.5 and 7.5 and the imidazolyl anion formed by the extraction of the two N–H atoms (Fig. 5 ▶). In our previous studies (Tanley, Schreurs, Kroon-Batenburg & Helliwell, 2012 ▶; Tanley, Schreurs, Kroon-Batenburg, Meredith *et al.*, 2012 ▶; Helliwell & Tanley, 2013 ▶) the overall summed occupancies of the two Pt atoms was greater than 100% and thus it was concluded that an imidazolyl anion was present rather than there being two tautomeric forms.

In the 65% MPD, 0.1 *M* citric acid crystallization conditions at pH 4.0, one would expect the His to be in its protonated form once again. Indeed, one week after crystal growth one molecule of carboplatin is seen to be bound to the N^δ^ atom (Fig. 4 ▶ *a*). Interestingly, when data were collected from a second crystal four weeks later, a molecule of carboplatin was seen to be bound to both the N^δ^ and N^∊^ atoms (Fig. 4 ▶ *b*). Since this crystallization condition contains citrate ions, the imidazolyl anion can again be formed, but since the citrate is at a much lower concentration compared with the Cl^−^ ions used previously (0.1 *M* compared with 1.4 *M*) we can expect the anion to form more slowly. Also, owing to the summed occupancy being less than 100% for these two Pt atoms (Table 2 ▶), histidine tautomers could be a possibility.

The crystals in 2 *M* ammonium formate, 0.1 *M* HEPES pH 7.5 grew quickly after a few days and the first X-ray diffraction data set was also collected fairly promptly, but no binding to His15 was seen (results not shown). However, when data were collected from a second crystal around 18 weeks after the initial crystallization was set up (Table 1 ▶) a molecule of carboplatin was seen to be bound to the N^δ^ atom of His15 (Fig. 4 ▶ *e*). At pH 7.5 the His residue exists in its deprotonated form; thus, only the other proton needs to be extracted to form the imidazolyl anion. This crystallization mixture contained a high concentration (2 *M*) of formate ions (HCOO^−^); thus, one can expect these formate ions to extract the second N–H atom, leaving two N atoms with lone pairs of electrons to bind to the Pt centre of carboplatin. Instead, we see a 2*F*_o_ − *F*_c_ electron-density peak 3.4 Å from the N^∊^ atom, and thus we cannot interpret it as a Pt atom even if it might be weakly bound owing to this unusually large distance. Also, no anomalous difference electron density is seen at this N^∊^ position either.

#### 4.2.2. The differing levels of structural detail seen for the carboplatin   

From all the crystal structure results, we see different amounts of detail in the N^δ^ and N^∊^ binding sites for the carboplatin molecule. The crystal at pH 5.6 shows the most detail in the N^δ^ binding site (Fig. 4 ▶ *d*), with more atoms of the CBDC moiety (Fig. 1 ▶) being modelled apart from the four-carbon ring structure, which still cannot be modelled owing to a lack of electron density (Fig. 4 ▶ *d*). A plausible explanation for not seeing this four-carbon ring structure is owing to dynamic disorder, as previously indicated in the small-molecule crystal structure of carboplatin determined by Beagley *et al.* (1985 ▶).

#### 4.2.3. Checks on the possible contamination of chloride ions from the HEWL lyophilized powder   

As the lyophilized HEWL powder still contains trace quantities of chloride ions, we naturally checked for any evidence of their binding. Thus, looking at the anomalous difference electron density of all the studies here down to 2.0σ possible signal levels, only the structure at pH 7.5 has any sign of such an anomalous peak, *i.e.* one peak of 2.5σ for just one of the usual chloride sites, whereas the other usual chloride-binding sites did not contain any anomalous peaks.

## 5. Conclusions   

Co-crystallization of HEWL and carboplatin in NaBr was carried out, with the XRD results showing that the carboplatin molecules indeed underwent a chemical conversion. This was to the transbromoplatin form, which contrasts with the NaCl case, where cisplatin was observed. Since a portion of the CBDC moiety was also still present, this confirms that the conversion of carboplatin to transplatin was partial (Tanley, Diederichs *et al.*, 2013*a* ▶).

Under non-NaCl crystallization conditions several of the HEWL crystals obtained led to carboplatin binding alone being observed. Of the four such crystal conditions obtained, the crystals grown in 0.1 *M* sodium citrate, 20% propanol, 20% PEG 4000 at pH 5.6 show the most detail that we have ever seen for the carboplatin molecule, with only the four-carbon ring structure not being present in the electron-density map.

### 6. Impacts and potential impacts   

Cisplatin and carboplatin are anticancer drugs which work by binding to the N7 atoms of guanine bases in DNA. Carboplatin is administered to patients and animals as a less toxic drug than cisplatin. The study involving the NaBr crystallization condition clearly confirms the partial chemical conversion of carboplatin to transbromoplatin. The previous study (Tanley, Diederichs *et al.*, 2013*a* ▶) under NaCl crystallization conditions showed partial chemical conversion of carboplatin to cisplatin. Carboplatin therefore has a frailty in its chemical behaviour under these chemical conditions. The question is whether carboplatin converts to transplatin or cisplatin en route during delivery to a tumour, presumably under saline conditions.

However, carboplatin can categorically preserve its chemical state under several chemical conditions that we have identified. Indeed, our crystal structure analyses for this set of chemical conditions, which may well be a subset of such chemical conditions, confirm its binding to histidine under these conditions. We have not categorically confirmed its entire, intact, chemical structure, as even in the most clearly defined crystal structure the end moiety of carboplatin was not resolved.

Apart from an anticancer role as chemical agents, carboplatin and cisplatin are used in combination therapy along with radiation therapy of tumours. It seems that these can be administered either after or before radiation treatment. If before, then maximal binding of the compound is sought in order to maximize X-ray absorption. With cisplatin and carboplatin binding to histidine residues, binding of cisplatin and carboplatin to any enzyme that uses histidine in its reaction mechanism could give added effects, beyond the accepted DNA binding mechanism of their action, in targeting tumour over normal cells.

## 7. Related literature   

The following reference is cited in the Supporting Information for this article: Helliwell (1988 ▶).

## Supplementary Material

PDB reference: HEWL co-crystallized with carboplatin in MPD conditions: crystal 1 processed using the *EVAL* software package, 4lt0

PDB reference: carboplatin binding to HEWL in NaBr crystallization conditions studied at an X-ray wavelength of 0.9163 Å, 4nsf

PDB reference: HEWL co-crystallized with carboplatin in MPD conditions: crystal 2 processed using the *XDS* software package, 4lt3

PDB reference: carboplatin binding to HEWL in 20% propanol, 20% PEG 4000 at pH 5.6, 4nsi

PDB reference: carboplatin binding to HEWL in NaBr crystallization conditions studied at an X-ray wavelength of 1.5418 Å, 4nsg

PDB reference: carboplatin binding to HEWL in 0.2 *M* ammonium sulfate, 0.1 *M* sodium acetate in 25% PEG 4000 at pH 4.6, 4nsh

PDB reference: carboplatin binding to HEWL in 2 *M* ammonium formate, 0.1 *M* HEPES at pH 7.5, 4nsj

Supporting Information.. DOI: 10.1107/S2053230X14016161/no5055sup1.pdf

## Figures and Tables

**Table 1:** X-ray crystallographic data and final protein model-refinement statistics for all crystals studied Values in parentheses are for the last shell.

PDB code	4nsf	4nsg	4lt0	4lt3	4nsh	4nsi	4nsj
Crystallization conditions	0.1*M* sodium acetate, 1*M* NaBr	0.1*M* sodium acetate, 1*M* NaBr	65% MPD, 0.1*M* citric acid buffer pH 4.0 crystal 1 (after one week)†	65% MPD, 0.1*M* citric acid buffer pH 4.0 crystal 2 (after five weeks)†	0.2*M* ammonium sulfate, 0.1*M* sodium acetate, 25% PEG 4000 pH 4.6	0.1*M* sodium citrate, 20% propanol, 20% PEG 4000 pH 5.6	2*M* ammonium formate, 0.1*M* HEPES pH 7.5
Apparatus	Diamond I04	Bruker APEX II	Bruker APEX II	Bruker APEX II	Bruker APEX II	Bruker APEX II	Bruker APEX II
Processing program	*MOSFLM*	*SAINT*	*EVAL*	*XDS*	*SAINT*	*SAINT*	*SAINT*
Time between crystallization setup and XRD data collection (weeks)	10	11	1	5	16	16	18
Data-collection temperature (K)	100	100	110	127	100	100	100
Data reduction							
Space group	*P*4_3_2_1_2	*P*4_3_2_1_2	*P*4_3_2_1_2	*P*4_3_2_1_2	*P*4_3_2_1_2	*P*4_3_2_1_2	*P*4_3_2_1_2
Unit-cell parameters ()							
*a* = *b*	78.58	78.37	76.77	77.12	77.08	77.71	77.49
*c*	37.29	37.21	36.36	36.56	37.14	36.84	37.12
Crystal-to-detector distance (mm)	205.7	40	40.2	40.4	40	40	40
Observed reflections	197352	150324	202135	206299	145058	294049	831022
Unique reflections	18627	8110	7770	7850	6965	9485	13055
Resolution ()	18.141.47 (1.501.47)	30.892.00 (2.102.00)	19.182.00 (2.032.00)	39.412.00 (2.052.00)	30.692.10 (2.132.10)	33.292.30 (2.652.30)	33.481.70 (1.801.70)
Completeness (%)	91.7 (64.2)	97.8 (85.9)	99.8 (99.8)	99.4 (32.4)	99.8 (99.7)	94.8 (100)	99.7 (98.7)
*R*_merge_ (%)	0.090 (0.460)	0.224 (0.619)	0.340 (2.71)	0.248 (2.27)	0.126 (0.499)	0.295 (0.505)	0.212 (0.942)
*I*/(*I*)	16.3 (4.2)	10.8 (1.3)	9.2 (0.9)‡	14.6 (0.5)§	19.2 (2.2)	7.5 (1.5)	19.3 (1.4)
Multiplicity	10.6 (8.9)	18.1 (3.6)	26.1 (12.3)	26.3 (6.0)	20.8 (5.8)	7.8 (7.2)	63.5 (30.1)
CC_1/2_			0.38	0.32			
Refinement							
Cruickshank DPI ()	0.07	0.27	0.25¶	0.19	0.30	0.60	0.13
Average *B* factor (^2^)	17.2	13.7	26.8¶	27.6	21.7	29.5	19.9
*R* factor/*R*_free_ (%)	12.5/18.1	21.8/27.0	22.3/28.3¶	19.5/25.7	19.6/26.1	22.0/28.4	20.0/25.9
R.m.s.d., bonds ()/angles ()	0.02/2.1	0.03/0.7	0.026/1.9¶	0.01/1.6	0.01/1.2	0.01/1.4	0.02/2.2
Ramachandran values (%)							
Most favoured	97.6	96.1	96.1	97.6	92.1	92.1	96.1
Additional allowed	2.4	3.9	3.9	2.4	7.9	6.3	3.9
Disallowed	0	0	0	0	0	1.6††	0

† These two data sets are described in an arXiv preprint (Tanley, Diederichs *et al.*, 2013*b* ▶); this preprint represented the start of the search for non-NaCl crystallization conditions for carboplatin with HEWL. Tanley, Diederichs *et al.* (2013*b* ▶) also logged ideas for combinations of criteria assessing diffraction data-resolution limits, the study of which is being pursued separately.

‡ *I*/(*I*) = 2.4 at 2.2, *I*/(*I*) = 1.7 at 2.15.

§ *I*/(*I*) = 2.17 at 2.17, *I*/(*I*) = 1.77 at 2.11.

¶ Final refinement statistics to 2.1 resolution for 65% MPD, 0.1*M* citric acid buffer pH 4.0 crystal 1 processed by *EVAL*, whereas the data-reduction statistics are to 2.0 resolution.

†† The two residues in the disallowed regions of the Ramachandran plot are Gly residues (Gly16 and Gly102).

**Table 2:** Anomalous difference electron-density peak heights for the Pt position at both the the N and N binding sites () along with the occupancy values of the Pt atoms (%) calculated using *SHELX* (Sheldrick, 2008 ▶)

	N		N		
	Anomalous peak height ()	Pt occupancy (%)	Anomalous peak height ()	Pt occupancy (%)	Summed occupancies
pH 4.0, crystal 1	5.1	39 2			39 2
pH 4.0, crystal 2	4.6	22 7	5.0	31 4	53 15
pH 4.6	9.5	49 8	5.2	40 8	89 11
pH 5.6, crystal 1	14.2	65 3	11.6	46 4	111 5
pH 7.5	5.1	29 3			29 3
NaBr, I04 Diamond	42.9	75 12	6.4	13 11	88 16
NaBr, Cu*K*	12.1	94 6	3.1	28 7	122 9

## Footnotes

1 Supporting information has been deposited in the IUCr electronic archive (Reference: NO5055).

## References

[bb1] Beagley, B., Cruickshank, D. W. J., McAuliffe, C. A., Pritchard, R. G., Zaki, A. M., Beddoes, R. L., Cernik, R. J. & Mills, O. S. (1985). *J. Mol. Struct.* **130**, 97–102.

[bb3] Calderone, V., Casini, A., Mangani, S., Messori, L. & Orioli, P. L. (2006). *Angnew. Chem. Int. Ed. Engl.* **45**, 1267–1269. 10.1002/anie.200502599 16416478

[bb4] Casini, A., Gabbiani, C., Mastrobuoni, G., Messori, L., Moneti, G. & Pieraccini, G. (2006). *ChemMedChem*, **1**, 413–417. 10.1002/cmdc.200500079 16892376

[bb5] Casini, A., Guerri, A., Gabbiani, C. & Messori, L. (2008). *J. Inorg. Biochem.* **102**, 995–1006. 10.1016/j.jinorgbio.2007.12.022 18289690

[bb6] Casini, A., Mastrobuoni, G., Temperini, C., Gabbiani, C., Francese, S., Moneti, G., Supuran, C. T., Scozzafava, A. & Messori, L. (2007). *Chem. Commun.*, pp. 156–158. 10.1039/b611122j 17180231

[bb7] Cianci, M., Helliwell, J. R. & Suzuki, A. (2008). *Acta Cryst.* D**64**, 1196–1209. 10.1107/S0907444908030503 19018096

[bb8] Cruickshank, D. W. J. (1999). *Acta Cryst.* D**55**, 583–601. 10.1107/s0907444998012645 10089455

[bb9] Dauter, Z. & Dauter, M. (1999). *J. Mol. Biol.* **289**, 93–101. 10.1006/jmbi.1999.2744 10339408

[bb10] Emsley, P., Lohkamp, B., Scott, W. G. & Cowtan, K. (2010). *Acta Cryst.* D**66**, 486–501. 10.1107/S0907444910007493 PMC2852313 20383002

[bb11] Fischer, S. J., Benson, L. M., Fauq, A., Naylor, S. & Windebank, A. J. (2008). *Neurotoxicology*, **29**, 444–452. 10.1016/j.neuro.2008.02.010 18439683

[bb12] Gust, R. & Schnurr, B. (1999). *Monatsh. Chem.* **130**, 637–644.

[bb13] Helliwell, J. R. (1988). *J. Cryst. Growth*, **90**, 259–272.

[bb14] Helliwell, J. R. & Tanley, S. W. M. (2013). *Acta Cryst.* D**69**, 121–125. 10.1107/S090744491204423X 23275170

[bb15] Huličiak, M., Vacek, J., Sebela, M., Orolinová, E., Znaleziona, J., Havlíková, M. & Kubala, M. (2012). *Biochem. Pharmacol.* **83**, 1507–1513. 10.1016/j.bcp.2012.02.015 22394404

[bb16] Kabsch, W. (2010). *Acta Cryst.* D**66**, 125–132. 10.1107/S0907444909047337 PMC2815665 20124692

[bb17] Kostova, I. (2006). *Recent Pat. Anticancer Drug. Discov.* **1**, 1–22. 10.2174/157489206775246458 18221023

[bb18] Leslie, A. G. W. (1999). *Acta Cryst.* D**55**, 1696–1702. 10.1107/s090744499900846x 10531519

[bb19] Lim, K., Nadarajah, A., Forsythe, E. L. & Pusey, M. L. (1998). *Acta Cryst.* D**54**, 899–904. 10.1107/s0907444998002844 9757106

[bb20] McCoy, A. J., Grosse-Kunstleve, R. W., Adams, P. D., Winn, M. D., Storoni, L. C. & Read, R. J. (2007). *J. Appl. Cryst.* **40**, 658–674. 10.1107/S0021889807021206 PMC2483472 19461840

[bb33] McNicholas, S., Potterton, E., Wilson, K. S. & Noble, M. E. M. (2011). *Acta Cryst.* D**67**, 386–394. 10.1107/S0907444911007281 PMC3069754 21460457

[bb21] Messori, L., Marzo, T., Gabbiani, C., Valdes, A. A., Quiroga, A. G. & Merlino, A. (2013). *Inorg. Chem.* **52**, 13827–13829 10.1021/ic402611m 24256441

[bb40] Murshudov, G. N., Skubák, P., Lebedev, A. A., Pannu, N. S., Steiner, R. A., Nicholls, R. A., Winn, M. D., Long, F. & Vagin, A. A. (2011). *Acta Cryst.* D**67**, 355–367. 10.1107/S0907444911001314 PMC3069751 21460454

[bb22] Schreurs, A. M. M., Xian, X. & Kroon-Batenburg, L. M. J. (2010). *J. Appl. Cryst.* **43**, 70–82.

[bb23] Sheldrick, G. M. (2008). *Acta Cryst.* A**64**, 112–122. 10.1107/S0108767307043930 18156677

[bb24] Tanley, S. W. M., Diederichs, K., Kroon-Batenburg, L. M. J., Schreurs, A. M. M. & Helliwell, J. R. (2013*a*). *J. Synchrotron Rad.* **20**, 880–883. 10.1107/S0909049513020724 PMC3795548 24121332

[bb25] Tanley, S. W. M., Diederichs, K., Kroon-Batenburg, L. M. J., Schreurs, A. M. M. & Helliwell, J. R. (2013*b*). http://arxiv.org/abs/1309.4661.

[bb26] Tanley, S. W. M., Schreurs, A. M. M., Helliwell, J. R. & Kroon-Batenburg, L. M. J. (2013). *J. Appl. Cryst.* **46**, 108–119. 10.1107/S0021889812044172 PMC3547227 23396873

[bb27] Tanley, S. W. M., Schreurs, A. M. M., Kroon-Batenburg, L. M. J. & Helliwell, J. R. (2012). *Acta Cryst.* F**68**, 1300–1306. 10.1107/S1744309112042005 PMC3515368 23143236

[bb28] Tanley, S. W. M., Schreurs, A. M. M., Kroon-Batenburg, L. M. J., Meredith, J., Prendergast, R., Walsh, D., Bryant, P., Levy, C. & Helliwell, J. R. (2012). *Acta Cryst.* D**68**, 601–612. 10.1107/S0907444912006907 22525758

[bb30] Weiss, M. S., Palm, G. J. & Hilgenfeld, R. (2000). *Acta Cryst.* D**56**, 952–958. 10.1107/s0907444900006685 10944331

[bb31] Winn, M. D. *et al.* (2011). *Acta Cryst.* D**67**, 235–242.

[bb32] Zhang, J.-G. & Lindup, W. E. (1996). *Toxicol. Lett.* **89**, 11–17. 10.1016/s0378-4274(96)03776-9 8952706

