# Re-refinement of 4xan: hen egg-white lysozyme with carboplatin in sodium bromide solution

**DOI:** 10.1107/S2053230X16000777

**Published:** 2016-02-19

**Authors:** Simon W. M. Tanley, Antoine M. M. Schreurs, Loes M. J. Kroon-Batenburg, John R. Helliwell

**Affiliations:** a School of Chemistry, Faculty of Engineering and Physical Sciences, University of Manchester, Brunswick Street, Manchester, M13 9PL, England; b Crystal and Structural Chemistry, Bijvoet Center for Biomolecular Research, Faculty of Science, Utrecht University, Padualaan 8, Utrecht, 3584 CH, The Netherlands

**Keywords:** 4xan re-refinement, raw diffraction data, paired model refinement, electron density assessment, resolution limit assessment, addendum

## Abstract

A re-refinement of 4xan, hen egg-white lysozyme (HEWL) with carboplatin crystallized in NaBr solution, has been made and is published here as an addendum to Tanley *et al.* [(2014), *Acta Cryst.* F**70**, 1135–1142]. This follows a previous re-refinement and PDB deposition (4yem) by Shabalin *et al.* [(2015), *Acta Cryst.* D**71**, 1965–1979]. The critical evaluation of the original PDB deposition (4xan), and the subsequent critical examination of the re-refined structure (4yem), has led to an improved model (PDB code 5hmj).

## Abstract 2

An addendum is published to Tanley *et al.* [(2014), *Acta Cryst.* F**70**, 1135–1142].

A re-refinement of 4xan, hen egg-white lysozyme (HEWL) with carboplatin crystallized in NaBr solution, has been made. This follows our response (Tanley *et al.*, 2015 ▸) to the critique article of Shabalin *et al.* (2015 ▸), suggesting the need for corrections to some solute molecule interpretations of electron density in 4xan and removal of an organic moiety as a ligand to the platinum ion coordinated to His15. This had been mistakenly included in our PDB file in an attempt by us to model the ‘shaped’ electron density for one coordination site to the Pt bound to the N^δ^ of His15, which we had rejected, and was not consistent with our Tanley *et al.* (2014 ▸) article. We have considered the preference of Shabalin *et al.* (2015 ▸) to model a chlorine in this density and a close-by bromine at partial occupancy to explain the ‘shape’. However, as the bromide concentration is in huge excess over chloride (by 20-fold), we think that the 4yem interpretation by Shabalin *et al.* (2015 ▸) is highly unlikely, but nevertheless we still cannot offer an explanation for that shape, confirming our earlier analysis described in Tanley *et al.* (2014 ▸).

The analysis presented here is based on new diffraction data processing to 1.3 Å resolution. The higher resolution limit was evaluated using *EVAL* (Schreurs *et al.*, 2010 ▸). In our accompanying *arXiv* article (Tanley *et al.*, 2016 ▸) we document in detail our different solvent and split occupancy side-chain electron-density interpretations as evidence for our statement of approach in our response article (Tanley *et al.*, 2015 ▸). Our critical re-examination includes comparisons based on 4xan diffraction data images that have been reprocessed with three different software packages so as to evaluate the possibility of variations in electron-density interpretations resulting from the use of different software. Overall our finalized model (PDB code 5hmj) (see Table S1 in the Supporting Information) is now improved over 4xan.

The following reference is cited in the Supporting Information for this article: Afonine *et al.* (2012 ▸).

## Supplementary Material

PDB reference: re-refinement of 4xan, 5hmj

Supporting Information.. DOI: 10.1107/S2053230X16000777/no5103sup1.pdf
