# Supplementary material for: Carboplatin binding to histidine
Source: Acta Crystallogr F Struct Biol Commun. 2014 Aug 29;70(Pt 9):1135–42. doi: 10.1107/S2053230X14016161 (PMC4157408; doi:10.1107/S2053230X14016161)
Supplement: Supplementary file 1 [file f-70-01135-sup1.pdf]

Acta Crystallographica Section F

Volume 70 (2014)

Supporting information for article:

Carboplatin binding to histidine

Simon W. M. Tanley, Kay Diederichs, Loes M. J. Kroon-Batenburg, Colin Levy, Antoine M. M. Schreurs and John R. Helliwell

Table S1 The 48 non-NaCl crystallisation screens from Hampton research.

10% MPD, 0.1M Citric Acid pH 4.0	0.1M NaAc, 2M NH_4sulfate
10% MPD, 0.1M NaAc pH5.0	0.2M NH4Ac, 0.1M Nacitrate, 30% MPD
10% MPD, 0.1M MES pH 6.0	0.1M NaHEPES, 1.4M Nacitrate
10% MPD, 0.1M HEPES pH 7.0	0.2M NH_4sulfate, 30% PEG4000
10% MPD, 0.1M NaAc pH 8.0	0.1M NaHEPES, 0.8M NaK tartrate
20% MPD, 0.1M NaAc pH 5.0	0.1M NaHEPES, 10% propanol, 20% PEG 4000
40% MPD, 0.1M Citric Acid pH 4.0	0.1M Nacacodylate, 1.4M NaAc
40% MPD, 0.1M NaAc pH5.0	0.5M $LiSo_4$, 15% PEG 8000
40% MPD, 0.1M MES pH 6.0	0.2M CaAc, 0.1M Nacacodylate, 18% PEG 8000
40% MPD, 0.1M HEPES pH 7.0	0.1M NaAc, 2M Naformate
65% MPD, 0.1M Citric Acid pH 4.0	0.1M NaHEPES, 2% PEG 400, 2M NH_4SO_4
65% MPD, 0.1M NaAc pH5.0	0.2M NH_4Ac, 0.1M NaAc, 30% PEG 4000
65% MPD, 0.1M MES pH 6.0	0.2M NaAc, 0.1M Nacacodylate, 30% PEG 8000
65% MPD, 0.1M HEPES pH 7.0	0.2M NH_4SO_4, 0.1M NaAc, 25% PEG 4000
0.1M NaAc, 2.0M NH_4SO_4	10%PEG 6000, 0.1M HEPES pH 7.5, 5% MPD
0.1M NaAc, 8% PEG 400	0.2M Nacitrate, 0.1M NaHEPES, 30% MPD
0.2M NH4Ac, 0.1M NaAc, 30% PEG 4000	0.4M NaK Tartrate
0.1M NaHEPES, 1.6M $NaKPO_4$	20% Jeffamine 500, 0.1M HEPES pH 7.5
0.1M Nacitrate, 1M NH_4PO_4	70% MPD, 0.1M HEPES pH 7.5
0.1M NaAc, 2M NaFormate	0.2M NH_4SO_4, 0.1M NaAc, 25% PEG 4000
0.1M imidazole, 1M 1M NaAc	2M NH_4 formate, 0.1M HEPES pH 7.5
0.2M Nacitrate, 0.1M NaHEPES, 20% propanol	0.2M NH_4SO_4, 0.1M NaAc pH 4.6, 30% PEG 2000
0.1M Nacitrate, 20% proponal, 20% PEG 4000	10% PEG 8000, 0.1M HEPES pH7.5, 8% Ethylene Glycol
0.2M Nacitrate, 0.1M Na cacodylate, 30% propanol	0.1M NaAc, 8% PEG 4000

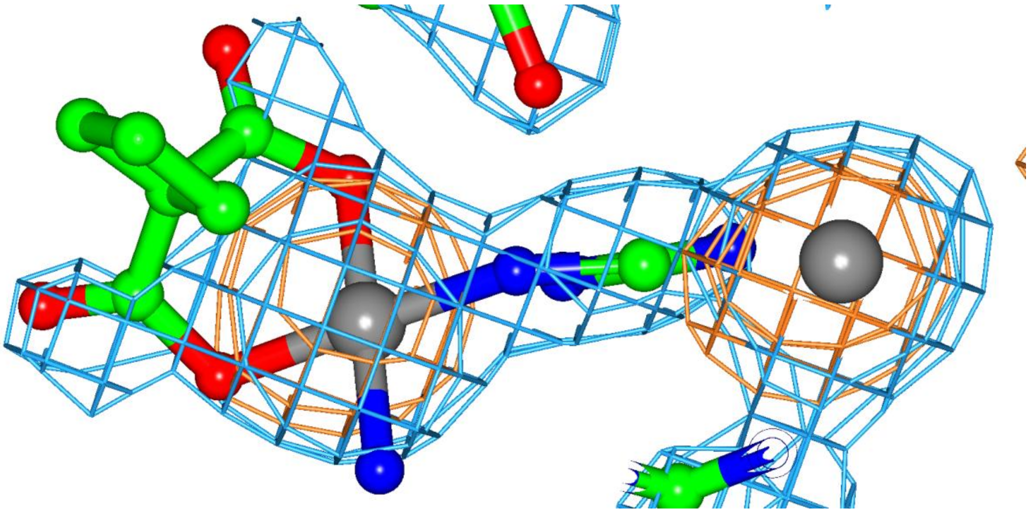

Figure S1 This figure shows the full carboplatin (from Beagley *et al*, 1985) superimposed on the electron density at the His15 binding site. It was from this that those carboplatin atoms not in electron density were deleted from the model. This figure can be compared directly with Figure 4d in the main text. 2Fo-Fc maps (blue) are shown at the 1.2rms contour level. Anomalous difference electron density (orange) maps are shown at the 3.0σ contour level. The platinum atoms are shown in grey, carbons are in green, oxygens in red and nitrogens in blue

S1. An unusual feature in the pH7.5 crystal structure at Cys 6-127

The dataset from the pH 7.5 crystal showed unusual 2Fo-Fc electron density as well anomalous difference density near one of the disulphide bridges Cys 6-127 (Figure S2). The distance between these two peaks of 3.4Å and their close proximity to the disulphide itself (3 to 4 Å) would suggest a reduced disulphide due to X-ray radiation damage (Helliwell, 1988). It did not prove possible to retain good polypeptide geometry and place each sulphur into the two peaks. As an additional check the first and last run of the XRD data collection was processed and the protein model refined separately to confirm whether this density was due to an X-ray radiation damage effect. However, the 2Fo-Fc electron density as well as the anomalous difference density is seen in the first data collection run as well as the last run. Another possible interpretation could be an alternative starting conformation of the disulphide bond. The crystallisation mix included HEPES buffer which contains sulphate ions. Thus, based on the anomalous difference electron density, the density might be due to two SO_4 ions, each partially occupied, as they would repel each otherwise.

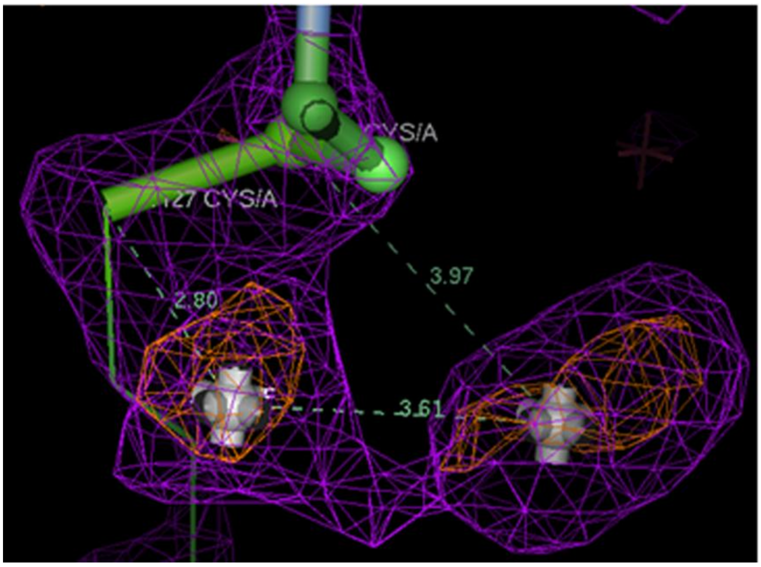

Figure S2 2Fo-Fc electron density as well as anomalous difference electron density nearby the Cys6-127 disulphide bond. Sulphur atoms are placed into the density to show the distances between the disulphide bond and these peaks.

Helliwell, J.R (1988) *Journal of Crystal Growth* **90**: 259-272
