# Supplementary material for: Re-refinement of 4xan: hen egg-white lysozyme with carboplatin in sodium bromide solution
Source: Acta Crystallogr F Struct Biol Commun. 2016 Feb 19;72(Pt 3):251–2. doi: 10.1107/S2053230X16000777 (PMC4774886; doi:10.1107/S2053230X16000777)
Supplement: Supplementary file 1 [file f-72-00251-sup1.pdf]

STRUCTURAL BIOLOGY
COMMUNICATIONS

Volume 72 (2016)

Supporting information for article:

Re-refinement of 4xan: hen egg white lysozyme with carboplatin in sodium bromide solution

Simon W. M. Tanley, Antoine M. M. Schreurs, Loes M. J. Kroon-Batenburg and John R Helliwell

Table S1 Crystal parameters, data processing and final model refinement statistics.

Statistics for the highest-resolution shell are shown in parentheses. The model refinement and validation software used was PHENIX_REFINE (Afonine et al 2012).

Wavelength (Å)	0.9163
Resolution range (Å)	39.29 - 1.30 (1.346 - 1.30)
Space group	P 4_3 2_1 2
Unit cell	78.58, 37.29
Total reflections	250129 (2024)
Unique reflections	23088 (889)
Multiplicity	10.8 (2.3)
Completeness (%)	78.76 (31.05)
Mean I/sigma(I)	10.86 (0.39)
Wilson B-factor (Å^2)	14.84
R-meas	0.095 (2.058)
$CC_{1/2}$	0.998 (0.181)
CC*	1.000 (0.553)
Reflections used for R-free	1170 (5.34%)
R-work	0.1642 (0.2946)
R-free	0.2150 (0.248)
Cruickshank 'Diffraction Precision Index' (Å)	0.061
Number of non-hydrogen atoms	1150
macromolecules	1039
ligands	29
water	82
Protein residues	129
RMS(bonds)	0.009
RMS(angles)	1.18
Ramachandran favored (%)	98
Ramachandran allowed (%)	2
Ramachandran outliers (%)	0
Average atomic *B* factor (Å^2)	18.3
B-factor Protein atoms (Å^2)	17.3
B-factor Ligand atoms (Å^2)	31.7
B-factor Solvent atoms (Å^2)	26.0
